# Navigating my career in lipid research

**DOI:** 10.1038/s41430-024-01452-6

**Published:** 2024-05-27

**Authors:** Andrew J. Sinclair

**Affiliations:** 1 Faculty of Health, Deakin University, Burwood, VIC 3125 Australia; 2 Department of Nutrition, Dietetics and Food, Notting Hill, VIC 3168 Australia

**Keywords:** Fatty acids, Biomarkers

## The start

My journey really didn’t start until I enrolled in a PhD in the Biochemistry Department at the University of Melbourne in 1965, although little did I know at the time that I would end up having a 55+ year career in nutrition. I chose to enrol for a PhD on essential fatty acids (EFA) although offerings in the Departments of Microbiology or Botany were equally of interest. One of life’s hindsight moments: why didn’t I investigate the merits of the projects in all three disciplines? My interest in biochemical metabolism was stimulated by the fascinating biochemistry lectures in the 3rd and 4th year of an Agricultural Science degree at the same university; this 4-year course covered a wide range of topics with the final year of eight subjects offering a wide range of career options for the graduates. My family connection to agriculture was through both my grandfathers (maternal grandfather owned an orange farm and my paternal grandfather was into agricultural education), though these links were not the reason I chose Ag Science – rather it was to do a course with lots of options after graduation. A highlight of my PhD time, aside from forming lifelong friendships with other graduate students, was being involved in a multidisciplinary investigation of the first case of EFA deficiency in an adult human [1].

My story is largely about multi-disciplinary collaborations and mentoring, a long, but extremely rewarding journey, having met so many talented, generous and mostly compatible people, many of whom are still friends. Speaking of friends, I must mention Robert (Bob) Gibson, my best friend, whom I have known since 1976; we have researched in complimentary areas, and it has been such a pleasure to chat to him over the years about nutrition, lipids, football and many other matters. How lucky are we to have had this long connection. Together we ran two very successful international fatty acid congresses which drew many visitors to Adelaide in 1992 [2] and to Cairns in 2006 [3].

I cannot pretend that what follows is a coherent pattern of research, apart from working mostly in the field of lipids, but rather it is a blend of goal-oriented research as well as a series of opportunistic endeavours often based on what funding opportunities were available.

## Postdoc positions – setting up an interest in arachidonic and docosahexaenoic acids

After an unremarkable postdoctoral position in London, Ontario, Canada, I was fortunate to spend four very interesting years at the Zoological Society of London’s Nuffield Institute of Comparative Medicine (NICM) (London, UK) in the period 1971–1974. NICM was a multidisciplinary institute with departments of Biochemistry (Michael Crawford), Haematology (Christine Hawkey), Pathology (Richard Fiennes) and Immunology (Alistair Voller). As an aside, it was a great opportunity to take our daughter to the London Zoo as often as she wished. Working as a postdoc in Michael Crawford’s lab was invigorating with the emphasis on polyunsaturated fatty acids (PUFA) in the brain, and in particular on arachidonic acid (AA) and docosahexaenoic acid (DHA). A major outcome from the team was the report that the brains of more than 30 different mammals, including humans, were rich in DHA (a long chain ω-3 fatty acid) [4] and this was at a time when most nutritionists did not regard the ω-3 FA as essential for humans. Additionally, we showed that human milk contained long chain PUFA, including AA and DHA [5]; and furthermore, that in rodent studies AA and DHA were rapidly incorporated into the brain compared with their precursors (linoleic acid (LA) and alpha-linolenic acid (ALA), respectively) [6]. In a study with NICM pathologist, Dick Fiennes, we reported the first case of ω-3 deficiency in a primate [7]. John Rivers was one of many colourful characters who worked in the Crawford group, and he found that domestic cats showed minimal metabolism of LA and ALA to their respective longer chain metabolites (AA and DHA) [8], which was later revealed to involve a novel pathway of PUFA metabolism. The implications were that carnivores required AA and DHA in their diets because the endogenous synthesis of these in the liver from LA and ALA, respectively, was very limited, a situation somewhat analogous to the requirement for retinol in carnivores rather than beta-carotene.

## Veterinary biochemistry, indigenous foods and lean red meat studies

Moving back to Australia in 1975, saw me take a position as a Research Scientist in the Victorian Department of Agriculture’s (DA) Veterinary Research Institute in Parkville from 1976 to 1985. The main focus of this period was veterinary diagnostic biochemistry, toxicology and haematology which involved regular discussions with the veterinarians and regional biochemists in veterinary DA laboratories throughout Victoria. During this time, we published papers on mannosidosis in Angus cattle [9], globoid cell leukodystrophy in a Polled Dorset sheep [10], zinc toxicity in ferrets [11] and establishment of methods to detect illegal meat-substitution in the food supply [12]. It was in this time that I started a research collaboration on the metabolism of PUFA in cats with Jock McLean (veterinary biochemist) [13], and, separately, a collaboration with Kerin O’Dea (a nutritional physiologist) on the identification of PUFA in Australian aboriginal/indigenous bush foods [14], which included the discovery of high proportions of AA in fish from coastal waters in northern Australia [15]. The findings of relatively high levels of AA in traditional animal foods and fish used by Australian aborigines led us to question whether AA-rich foods were prothrombotic, as suggested by the literature [16].

In 1985, I moved to RMIT University in Melbourne, and with Kerin O’Dea we continued a series of intervention studies of diets rich in Aboriginal bush foods including fish from northern Australian coastal waters, and kangaroo meat compared other foods such as n-3 rich fish, or lean white meat, with the objective of examining whether foods rich in AA were prothrombotic. We studied bleeding times, blood lipid levels, plasma fatty acids and eicosanoid production [17, 18]. These studies were supported by investigations in rodents on effects of AA and EPA on thromboxane and prostacyclin production [19]. The results of human studies revealed that diets that contained both AA and long chain ω-3 FA were associated with an increase in prostacyclin production, without affecting thromboxane production, supporting the idea that foods which contained AA were not prothrombotic [20].

In the course of this work, we became interested in the nutritional properties of lean red meat (from grass-fed beef cattle, grass-feeding being a typical practice in Australia). We demonstrated that 500 g lean beef/day did not raise blood cholesterol levels [21]. Subsequent studies revealed that lean beef contained >50 mg of long chain ω-3 FA/100 g raw weight [22] and that consumption of 500 g lean beef/day was associated with an improvement of plasma ω-3 FA profile, especially EPA and DPA [23]. Meat and Livestock Australia (MLA) were particularly interested in the ω-3 story in beef and lamb and were keen to have these food sources listed as a good source of ω-3 FA. MLA encountered difficulties related to claiming DPA as source of ω-3 FA. Ruminant meats, including beef and lamb, contained DPA ω-3 for which the food standards authorities claimed there was no evidence to support this FA to be included in an ω-3 claim. This led to MLA support for our group to study the nutritional properties of DPA ω-3 (I will have more to say on this later).

## Food science, haematology, visual function and alpha-linolenic studies (meat and veg)

In 1995 (until 2006), I joined a new Department of Food Science at RMIT University and embarked on three major collaborations including: [a] diet and thrombosis with a haematology team led by Alan Turner (dec.), Neil Mann (nutritionist) and Duo Li (physiologist/chemist); [b] physiological role of alpha-linolenic acid on ingestive behaviour, blood pressure and thirst with physiologist Richard Weisinger, optometrist Harry Weisinger and psychologist Denovan Begg, and [c] the effect of ω-3 FA deficiency on visual function with optometrist and optical measurement expert Algis Vingrys. In each area we were fortunate to have outstanding students from different backgrounds and disciplines who contributed significantly to the programmes. In addition to these collaborations, studies were also conducted by our group on ALA content of green leafy vegetables [24] and the ALA deposition in tissues leading to the identification of skin and fur as major sites for ALA deposition in guinea pigs and rats [25]. This work remains as unfinished business (Table 1). A review on the roles of ALA in mammals was written during this phase of ALA research [26].

**Table 1:** Unfinished business.

Fatty acid	Area of study
ALA	What is the role of ALA and/or ALA-lipid mediators in skin and fur/hair?
AA	What is the role of AA-derived lipid mediators in learning? How are these mediators influenced by ω-3 deficiency?
DPAω-3	Exploring biological roles of DPAω-3. Does DPA reduce post-prandial lipaemia? What is DPA role/function in brain?
DHA and zinc	Exploring the biological significance of the in vivo relationship between DHA and Zn.
CMPF	Is CMPF a long chain ω-3 metabolite? If so, which specific ω-3 is the precursor? To what extent does CMPF account for physiological properties of ω-3 FA?
Red blood cells^a^	How do red blood cells acquire their ω-3 FA?

*ALA* alpha-linolenic acid, *AA* arachidonic acid, *CMPF* 3-carboxy-4-methyl-5-propyl-2-furanpropanoic acid, *DHA* docosahexaenoic acid, *DPAω-3* docosapentaenoic acid ω-3, *FA* fatty acid, *Zn* zinc.

^a^This is a new project.

The diet and thrombosis team were especially interested in the effects of vegetarian and vegan diets compared with diets containing meat on platelet aggregation and thrombotic factors. In addition, we studied the effects of dietary components such as saturated fat, gamma-tocopherol and cocoa flavanols on platelet aggregation and thrombotic factors, with these studies involving dietary interventions in human subjects for periods of two to four weeks. Somewhat surprisingly, vegans compared with meat eaters had increased platelet aggregation and platelet volume [27], and increased plasma homocysteine levels [28]. Lower platelet aggregation was observed following the ingestion of cocoa powder [29] and gamma tocopherol [30], compared with placebos. In separate studies, we showed that it was possible to increase platelet levels of palmitic and stearic acids, respectively, after diets enriched in these FA [31]. Platelet aggregation was increased on the palmitic acid-enriched diet in comparison with the stearic acid-enriched diet [32], suggesting options for the food industry to use stearic acid to replace trans fatty acids in manufactured foods. It is such a pity that at the time we had not formed any collaborations in the lipidomics field to investigate the lipid molecular species changes in the platelets.

A major focus of the ingestive physiology studies was the effect of ω-3 deficiency on blood pressure in rats. We found that perinatal ω-3 deficiency was associated with raised blood pressure later in life (in rats), independently of whether ω-3 FA were provided after the deficient perinatal period or not [33]. Further studies reported that there was an interaction between ω-3 deficiency and the level of dietary protein on hypertension [34], with mechanistic investigations leading to the conclusion that the hypertension resulting from ω-3 deficiency was mediated by the central adrenergic system [35]. During the course of working with Richard Weisinger and Denovan Begg, aged ω-3 deficient rats were found to have significant alterations in brain arachidonic acid (ω-6 fatty acid) metabolism, including significantly elevated hypothalamic PGE(2) [36]. These novel observations were followed up by testing the behavioural effects of refeeding ω-3 deficient animals with either ω-3 FA or a COX inhibitor. Treatment with either ω-3 FA or a COX inhibitor prevented spatial-recognition deficits in 3^rd^ generation of ω-3 deficient mice, suggesting cognitive impairment due to dietary ω3 FA deficiency may be related to inflammation arising from the arachidonic acid-COX pathway [37]. This work is also unfinished business, although it is known that COX-2 is an essential component in memory formation [38] (Table 1).

The visual function of rats and monkeys is functionally dependent on ω-3 FA, based on studies in ω-3 deficient animals [39, 40]. The role of the ω-3 FA is specifically related to DHA-phosphoglycerides being intimately involved in visual transduction process (these phosphoglycerides allowing the expansion of rhodopsin following the incident photon of light) [41]. In 1986, it was reported that the visual function of guinea pigs was unaffected by ω-3 deficiency [42], and since this was not consistent with the role of DHA in the retina as reported in other species, I decided to repeat this work, and established a visual function group in collaboration with vision expert, Algis Vingrys. We showed firstly that the visual function of guinea pigs was highly dependent on a dietary supply of ALA [43]; secondly that optimum visual function was associated with retinal DHA in an inverted U-shaped curve response [44]; and thirdly that visual function was significantly compromised, once more than a quarter of the DHA had been lost from the retinal lipids as a result of ω-3 deficiency [45]. This collaboration lasted for more than 10 years, perhaps cemented because our laboratories were only 20-min apart involving walking through Carlton, a very Italian area of Melbourne with many cafes.

## Zinc and ω-3 connection

In this period, another important collaboration was established with two Hungarian molecular biologists, Laszlo Puskas and Klara Kitajka, on the effects of ω-3 FA on brain gene expression. Firstly, it was shown that provision of ω-3 FA led to a formidable range of genes being impacted, including those involved with synaptic plasticity, cytoskeleton, signal transduction, ion channel formation, energy metabolism and regulatory proteins [46]. A second study using ω-3 deficient rats unexpectedly showed that the deficient animals had increased expression of ZnT3 (a zinc transporter) in the brain and this was associated with an increased level of free zinc in the hippocampus [47].

The work on zinc and ω-3 FA continued with biochemist Leigh Ackland and immunologist Cenk Suphioglu, this time in human neuronal cells, once I moved to Deakin University in 2006. It was found that DHA could ablate the deleterious effects of added zinc on these cells. In response to increase in zinc concentration, increased deacetylation, methylation and phosphorylation of histone H3 and increased apoptosis was observed [48]. Conversely, DHA treatment resulted in decreased deacetylation, methylation, phosphorylation of histone H3 and decreased caspase-3 levels [49], suggesting that DHA promoted gene expression and neuroprotection. These novel findings highlighted the potential benefit of DHA for management of neurodegenerative diseases. More research needed here (Table 1).

## Diversifying: using lipidomics to differentiate effects of dairy v soy diets, krill oil v fish oil and DPA v EPA on molecular species of lipids

In 2006, I moved to Deakin University (School of Exercise and Nutrition Science in Melbourne) and then in 2010 to the Metabolic Research Unit, School of Medicine, Deakin University (Geelong). There were many productive collaborations during this time, always involving interesting and talented graduate students. Through a collaboration with Peter Meikle (biochemist), we were fortunate to be able to employ comprehensive lipidomic analysis of plasma and chylomicron lipids in three postprandial intervention studies (dairy versus soy; DPA versus EPA; krill oil versus fish oil) [50–52] and a 30-day dietary supplementation study (krill oil versus fish oil) in collaboration with Xiao Su (biochemist). This technology provided details of the molecular species of lipids with a highly significant increased sensitivity compared with gas chromatography. Two examples which stood out were the identification of plasmalogens in plasma being significantly more labelled with ω-3 FA after krill oil compared with fish oil [53], and the distinctly different molecular species of chylomicron TAG’s which DPA was incorporated into compared with EPA [51].

## DPA ω-3 studies finally

During this time, we conducted several studies on DPA ω-3, both in animals and in humans as we had gained access to pure DPA ω-3 in a sufficient quantity to conduct short-term intervention studies, with David Cameron-Smith (exercise physiologist), chemist (Peter Meikle), molecular biologist (Juan Molero), food scientist (Kaisa Linderborg), and nutritionist (Duo Li). These studies revealed that DPA administration/supplementation led to the rapid appearance of significant proportions of DPA and also of EPA in cell culture and rats [54] and humans [55], however increases in DHA were not consistently observed. The extent of metabolism of DPA ω-3 to EPA had not been appreciated previously. Two novel findings resulted from the 7-day supplementation study were firstly that DPAω-3 resulted in a significantly lower postprandial lipaemia compared with EPA [51] and secondly that specialised pro-resolving lipid mediators (SPM) of DPA (resolvin and maresin) were identified in plasma following 7-days of DPA supplementation [56]. Another novel finding, in rats, was that very little of orally dosed 14C-DPA and 14C-DHA were metabolised to CO2 over a 6-hr period compared with 14C-EPA, consistent with greater muscle deposition for DPA & DHA [57]. In a separate study conducted with colleagues in China (Duo Li), pure DPA, DHA and EPA were provided to volunteers for a short time and we showed a strong correlation between plasma DPA and 3-carboxy-4-methyl-5-propyl-2-furanpropanoic acid (CMPF), which is a furan fatty acid metabolite, but which may also be an ω-3 metabolite [58]. There are still many unanswered questions about the physiological role of DPA ω-3 [59], however it does seem that is a precursor of EPA and also of its own unique SPM’s (Table 1).

## ω-3 Bioavailability studies

Prior to my retirement from Deakin University, I started a collaboration with an expert fish nutritionist and lipidologist, Giovanni Turchini, on matters related to bioavailability of long chain ω-3 FA (PLR) using the whole-body fatty acid balance method [60]. After a thorough review of the literature on ω-3 FA bioavailability [61], we conducted a bioavailability study in rats, where we found that there were differences in absorption of fish and krill oil between male and female rats; that most of the EPA and just over half of DHA were β-oxidised in both diet groups, and that significantly more DPA and DHA were deposited in tissues in the krill oil group [62]. The unexpected findings of the second study showed that a large dose of ω-3 LC-PUFA fed once per week was more effective in increasing whole body ω-3 LC-PUFA content in rats compared with the same total amount of the dose delivered daily [63].

## Retirement: a stint China

After retirement, I worked in China for about 12 months, in teaching and research at Zhejiang University (Hangzhou) and South China University of Technology (SCUT, Guangzhou). This was a thoroughly enjoyable time, meeting so many enthusiast and capable students and my only regret was that I had not worked in China earlier in my career nor learned to speak mandarin. A major output was to write a review on furan fatty acids [64] with some really talented graduate students in Prof Wang Yonghua’s laboratory at SCUT. This led to an interest in CMPF which is a metabolite of both furan fatty acids and a putative metabolite of long chain ω-3 FA as referred to earlier. CMPF is proving to be an interesting compound and has been associated with a lower risk of type 2 diabetes in humans [65]. More research is needed here (Table 1).

## Crystal ball

I thought I would begin with my reflections on the current state of nutrition. We shouldn’t forget this field came about because of close collaborations between chemists, physiologists and medical doctors and today is it patently obvious that many important works in the field also arise through collaborations of various sorts: food structure (physics/chemistry), sensory science (chemistry/physiology), microbiome studies (microbiologists/physiologists/immunologists), various ‘omics research studies (chemistry/biochemistry/bioinformatics), epidemiology (statistics/public health) and so on. Further to this, there are nutrition-related studies involving close collaborations with experts in bone health, respiratory function, cardiovascular health, mental health, and so on. Clearly it is not possible to train undergraduate nutrition scientists in all fields, but they should recognise that their future careers in research will likely depend on them forging relationships with specialists in other fields. My prescription for a nutrition science degree is a solid foundation in chemistry, physiology, biochemistry, statistics, research design and methods and systematic reviews, behavioural psychology, critical thinking, cutting edge technologies, agriculture and the food chain and the adaption to climate change, and the influence of AI in all these fields. In addition, given the way the world’s climate is changing so rapidly, I believe all graduates in nutrition (and other allied health courses) should have basic training in first aid, disaster relief skills, crisis management, the effects of heat on health, and some knowledge about keeping infants and the elderly alive in a crisis. Where possible, these trainings should be integrated into the course materials.

## Final reflections

I have found it both valuable and rewarding to be involved for so long in multidisciplinary research. Aside from the benefits to the projects, the friendships which frequently developed added to the overall rewards of being involved in research, which is often a hard slog. Another issue, I place great store in is mentoring students. This can be extremely challenging at times, but I have found it stimulated me to think of ways to interact with the many different personalities that we come across in the research. As supervisors/trainers, we have to get along with the students to get the best out of them for their sake and also for the sake of the project, which ultimately is what we are all interested in. An enduring passion of mine has been my involvement with the Nutrition Society of Australia (NSA) since 1975. It has been such a pleasure to see this organisation grow from strength to strength over this time and for me to have maintained long-term friendships with NSA members.

Where is the field moving? In former times, nutrition could be thought of as ‘spray and weigh’ and maybe measuring a couple of systemic biological markers, however it has become increasingly complex with the ability to apply a wide range of highly sophisticated techniques, including ‘omics, imaging techniques and complex statistical methods to study the impact of particular treatments/interventions. Such technologies make the interpretations increasingly difficult and typically require the support of bioinformatics/statistical experts in the research team. This leads to the difficulties faced by researchers in university departments where newly appointed lecturers have one graduate student and one relatively small project with a limited budget. With the emphasis on the need for publications to progress and to graduate PhD students, small teams face significant challenges, and often new staff are recommended to form alliances with other groups. This is good advice; however, it does not guarantee sufficient funds to investigate the research issues using the latest, typically expensive, techniques. So, life can be extremely challenging for young staff.

Finally, it is only since I retired from paid work that I have found time to think and write some reviews [66, 67]. Pre-covid, thinking time could rarely be accomplished at work and it was only by taking a day off or on weekends or late nights that this might be possible. However, since covid, more and more time can be spent away from the office/lab so quiet thinking time has become a more realistic possibility. In addition to heavy workloads and busy lives, achieving a work-life balance and having a supportive family are just as important for nutrition scientists as those in other professions. As scientists we are so fortunate that our research often becomes a hobby which makes life so interesting, however we should never forget that teaching is the fundamental responsibility of an academic. I want to finish with one question which really helps to ground oneself when everything looks really bleak (didn’t get the grant, paper rejected etc), and that is ‘what are we here for?’. When everything seems to weigh heavily on one’s shoulders, I have found reflecting on this question helps to keep oneself grounded.
